# Electroencephalogram (EEG) Based Prediction of Attention Deficit Hyperactivity Disorder (ADHD) Using Machine Learning

**DOI:** 10.1192/j.eurpsy.2025.1155

**Published:** 2025-08-26

**Authors:** J. Kim, S. Yang

**Affiliations:** 1 Psychiatry, Daegu Catholic University School of Medicine, Daegu, Korea, Republic Of

## Abstract

**Introduction:**

Attention Deficit Hyperactivity Disorder (ADHD) is a complex neurodevelopmental disorder that presents challenges in achieving accurate and timely diagnosis.

**Objectives:**

This study investigates the effectiveness of using electroencephalogram (EEG) data combined with machine learning techniques to enhance the prediction accuracy for ADHD diagnosis.

**Methods:**

A total of 168 subjects were evaluated using the Kiddie Schedule for Affective Disorders and Schizophrenia Present and Lifetime Version Korean Version (K-SADS-PL-K), categorizing them into two groups: ADHD (n=107) and Neurotypical (NT, n=61). We analyzed quantitative EEG (qEEG) data across 19 channels, focusing on frequency ranges including delta (1–4 Hz), theta (4–8 Hz), alpha (8–12 Hz), beta (12–25 Hz), high beta (25–30 Hz), and gamma (30–80 Hz). To classify ADHD versus NT groups, the Extreme Gradient Boosting (XGBoost) classifier was utilized, with Leave-One-Subject-Out (LOSO) cross-validation employed to assess model performance.

**Results:**

To address the issue of limited data, we segmented each subject’s EEG data into 30-second intervals, resulting in 2434 segments for the ADHD group and 1060 segments for the NT group (figure 1). These segments were used to train the machine learning model. The XGBoost algorithm, combined with the LOSO cross-validation strategy, achieved a test accuracy of 90.81% and an F1 score of 0.9347, demonstrating robust performance in distinguishing between ADHD and NT subjects (figure 2). Feature importance analysis using SHAP values highlighted that EEG features from specific frequency bands, particularly the middle beta/theta ratio and relative gamma of O1 electrode sites, played a crucial role in the classification (figure 3).

**Image 1:**

**Figure:**
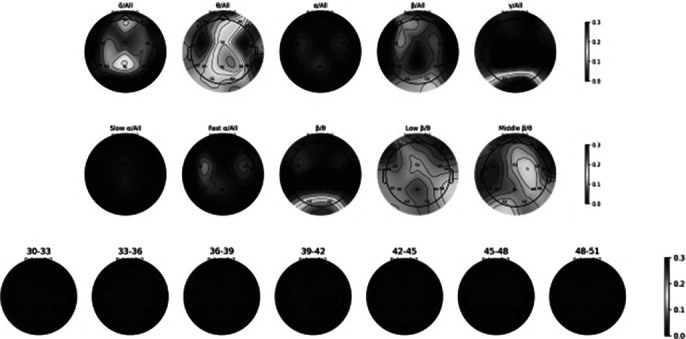

**Image 2:**

**Figure:**
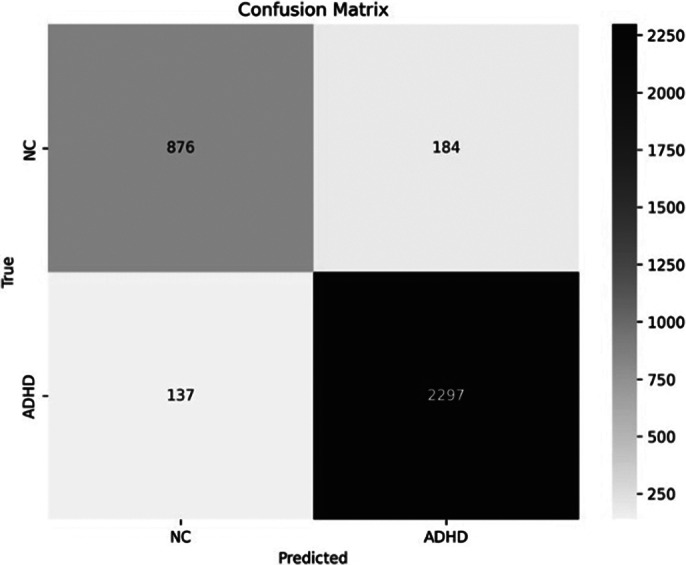

**Image 3:**

**Figure:**
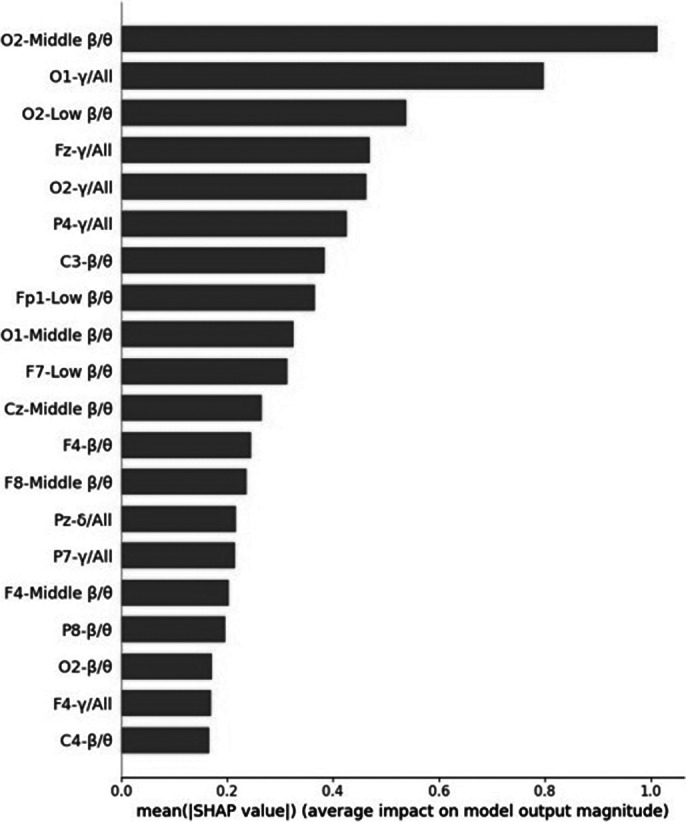

**Conclusions:**

Our findings suggest that EEG-based machine learning models hold significant potential as non-invasive tools for assisting in the diagnosis of ADHD. Further research with larger datasets and additional validation is necessary to confirm these results and explore their clinical applicability.

**Disclosure of Interest:**

None Declared

